# Population genetic models for the spatial spread of adaptive variants: A review in light of SARS-CoV-2 evolution

**DOI:** 10.1371/journal.pgen.1010391

**Published:** 2022-09-22

**Authors:** Margaret C. Steiner, John Novembre

**Affiliations:** 1 Department of Human Genetics, University of Chicago, Chicago, Illinois, United States of America; 2 Department of Ecology & Evolution, University of Chicago, Chicago, Illinois, United States of America; University of Rochester, UNITED STATES

## Abstract

Theoretical population genetics has long studied the arrival and geographic spread of adaptive variants through the analysis of mathematical models of dispersal and natural selection. These models take on a renewed interest in the context of the COVID-19 pandemic, especially given the consequences that novel adaptive variants have had on the course of the pandemic as they have spread through global populations. Here, we review theoretical models for the spatial spread of adaptive variants and identify areas to be improved in future work, toward a better understanding of variants of concern in Severe Acute Respiratory Syndrome Coronavirus 2 (SARS-CoV-2) evolution and other contemporary applications. As we describe, characteristics of pandemics such as COVID-19—such as the impact of long-distance travel patterns and the overdispersion of lineages due to superspreading events—suggest new directions for improving upon existing population genetic models.

## Introduction

The Coronavirus Disease 2019 (COVID-19) pandemic has been one of the most significant events in recent human history where the processes of evolutionary biology are unquestionably paramount. The importance of “variants of concern” (VOCs) is now well recognized, and substantial effort now goes to monitoring and studying their properties [1,2]. In considering any adaptive variant, one of the key aspects of its evolutionary dynamics is how it spreads geographically, from the place of its origin to populations potentially across the globe. In the context of COVID-19, the successive establishment and geographic spread of adaptive variants has become a major factor in the progression of the pandemic and is now a dominant management challenge in reacting to and quelling the pandemic. Intrinsic to this process is the geographic spread of an adaptive variant, a topic that has long been studied in evolutionary population genetics using theoretical models.

Motivated by COVID-19 and the dispersal of variants of infectious agents more broadly, we provide a review of the theoretical population genetic literature on models for the geographic spread of adaptive alleles. While this has been an ongoing area of research for over 80 years, no recent literature review of these models is readily available. In our writing, we give special attention to how relevant these models are to the problems occasioned by the spread of adaptive variants in pathogens. In a retrospective way, we ask: Given this long history of study, were the theoretical models available as the pandemic began ready to provide insights regarding Severe Acute Respiratory Syndrome Coronavirus 2 (SARS-CoV-2)? And to the extent they were not, what gaps exist and what research directions should be emphasized for the future?

While we limit ourselves to the theoretical population genetic literature, evolutionary aspects of pandemics overlap with many academic disciplines, and we recommend readers also see other excellent reviews in this broad area (for instance, [3–5]). We additionally limit our scope to prospective, forward-in-time theoretical population genetic models, thus excluding retrospective approaches such as genealogy-based and phylogeographic models for which existing reviews are available (see [6,7], respectively). As we will show, the COVID-19 pandemic highlights several gaps in current models for the geographic spread of adaptive alleles, the resolution of which will be informative for both scientific and public health goals.

**Fig 1:**
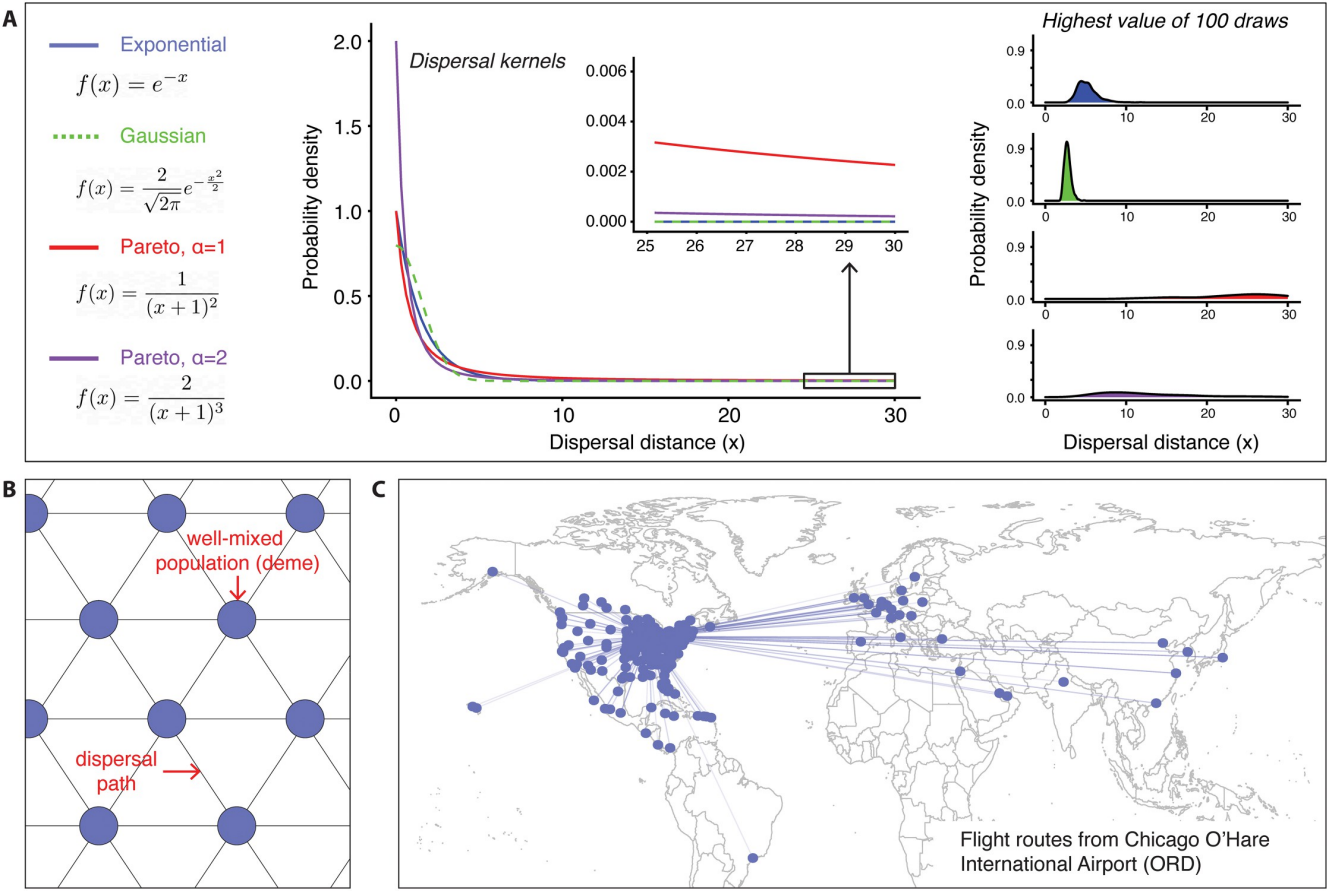
(A) Negative exponentially bounded vs. fat-tailed dispersal kernels. (Left) Four example dispersal kernels: exponential, folded Gaussian, and Pareto distributions. The Pareto distribution is a form of power-law distribution, which is a classic example of a “fat-tailed” distribution. (Middle) Probability density of each dispersal kernel, with the inset showing values in the tail. (Right) Density plots obtained from the set of highest values of 100 draws for each dispersal kernel. (B) Nearest-neighbor stepping stone model of migration. (C) Example of non-nearest-neighbor migration in the form of commercial flight routes originating from O’Hare International Airport in Chicago, Illinois. Dots represent airport locations. Constructed using publicly available data from https://openflights.org/data.html and Natural Earth using the R package ggmap [9].

Before reviewing specific theoretical models of spatial spread with selection, it is necessary to introduce some foundational vocabulary for each of the processes involved in the spatial spread of alleles. At its core, dispersal involves movement of individuals between locations in *space*, as described by either *continuous* or *discrete* spatial models. In many continuous models, dispersal is assumed to be *diffusive*, meaning, dispersal is dominated by short-range movement with few to no large, discontinuous jumps. Alternatively, when large, discontinuous jumps are more common, dispersal is described as *fat-tailed*. The name arises because if one considers a probability distribution on the geographic displacement between an offspring allele and its parental allele (Fig 1A; also known as a *dispersal kernel*), the distribution has substantial probability mass in its tails, which represent long-distance jumps. Formally, the tails decay slower than an exponentially decaying function (Fig 1A; and see [8] for more on dispersal kernels). Dispersal may be *isotropic*, meaning movement in any direction is equally probable or *anisotropic* (for example, when movement occurs along predominant axes). Lastly, dispersal may also be pairwise *symmetric* or *asymmetric*, an important example of asymmetry being where dispersal has a nonzero displacement vector (as might arise when movement in one direction is greater than in the reverse direction). Dispersal can also be spatially *homogeneous*, meaning the same dispersal distribution applies across the whole space, or in more complicated cases, spatially *heterogeneous*. In some cases, dispersal is modeled as occurring among discrete populations (for example, lattice, stepping-stone, meta-population, and network models; Fig 1B). In these models, locations take the form of nodes in a *network* of discrete units, typically representing local well-mixed subpopulations, known as *demes* in the population genetic literature (Fig 1B). In this case, varying numeric weights on the edges connecting individual demes can be used to model spatially heterogeneous levels of dispersal, and the presence of edges between distant nodes in the network can represent long-distance dispersal (Fig 1C).

In addition to dispersal, one must consider basic features of the mutational, adaptive, and reproductive processes. First, in the simplest case, an adaptive variant can be traced back to a single mutational event (variously called a *unique event polymorphism* or that all carriers of the mutation are *identical-by-descent*). Alternatively, in scenarios with *multiple origins* or *recurrent mutation*, a particular mutation may have arisen multiple times, complicating the spatial modeling. The *adaptive sequence landscape* is a mapping of sequences to fitness, with an important feature being how many single-mutant neighbors of a particular sequence result in an increase in fitness and by how much [10]. Notably, the adaptive landscape can vary both spatially and temporally as local conditions change. The fitness of an allele will impact the number of offspring its carriers will have, i.e., *the distribution of offspring number*. A key feature of the distribution of offspring number is its variance and the relative amount of density in the tail (i.e., skew). In classical population genetic (and epidemiological) models, the variance of offspring number is usually assumed to be finite (for example, Cannings models [11]) and sometimes assumed to be small (for example, in the Wright–Fisher model, approximately 1) and with the distribution having an exponentially bounded tail. Alternatively, in models with *overdispersed offspring number distributions*, a few carriers may have very large number of offspring, creating what are referred to as “superspreading” events in an epidemiological context [12].

An additional note of clarifying vocabulary is that the terms *variant*, *allele*, and *clade* have closely related and often overlapping meanings and uses. Many classic theoretical population genetic models are formulated in terms of the abstract notion of an *allele*, which denotes a form of genetic material in a particular locus, regardless of its exact molecular basis. The term *variant* is quite similar, though used more often in a modern context where the exact molecular basis of the allele, i.e., the defining mutation(s), is known. Phylogenetic approaches often classify variation in the form of *clades* within an inferred phylogenetic tree: Members of the same clade carry a shared set of mutations that occurred on branches ancestral to the node defining the clade. Models for the spread of an allele, in many cases, can be applied to the spread of a clade. Clades are also sometimes referred to as lineages. For instance, the Pango nomenclature system identifies *lineages* with epidemiological relevance [13,14]. Additionally, the Greek letter system used by the World Health Organization denotes *variants of concern* and *variants of interest* based on evidence of impact on disease characteristics (for instance, transmissibility; [15]). Both Pango and Greek letter lineages/variants are also related to clade definitions given by Nexstrain [2] and GISAID [16]. It is important to note that this nomenclature is not consistent across viruses, with HCV and HIV lineages being referred to commonly as “genotypes” and “subtypes,” respectively [17,18]. In this paper, given our intention to focus on the theoretical population genetics literature, we will often use the terms allele and variant, noting that clade in a phylogenetic tree is a special case of an allele where all carriers are identical by descent (see above).

Given the range of possibilities implied by the vocabulary just introduced, theoretical models can take many forms, with each conferring a degree of approximation or simplification. In this review, given our motivating interest in the geographic spread of SARS-CoV-2 variants, we focus mostly on the major landmarks in the spatial modeling of adaptive variant evolution and discuss relevant aspects of mutational and reproductive processes as they arise.

Before beginning, we need to clarify one more key aspect of the terminology in our writing. In the population genetic models we discuss, the processes of geographic dispersal, mutation, and reproduction each occur every generation. To think about these models in the context of a virus such as SARS-CoV-2, a natural simplification is to treat each passage from infection to transmission as a reproductive generation for the virus. In this simplification, any change in dominant viral type between an infection and transmission (i.e., within-host evolution) is considered as a mutation. Given most SARS-CoV-2 transmission occurs over a spatial scale of meters, the dispersal in each “generation” is primarily mediated by the movement of infected individuals. Additionally, in the case of SARS-CoV-2, the environment includes the immune system of the human host (as well as any other localized factors affecting transmission). Thus, the treatment of SARS-CoV-2 in the framing of these evolutionary models represents a substantial simplification. Yet, there have been few reviews of the theoretical population genetic models of spread and the lens of SARS-CoV-2 provides an interesting test case for understanding new directions in which the models could be developed.

## Population genetic models for the spatial spread of advantageous alleles: From the wave of advance model to recent developments

The natural starting point for models for the spread of adaptive alleles is the “wave of advance” model, which assumes instances of an allele (or a species) disperse geographically according to a diffusive motion and increase in frequency due to a selection pressure (or intrinsic growth rate) that is constant everywhere (also known as Fisher-KPP models; [19–21]). More formally, this model is described by a second-order partial differential equation, where *p*(*x*, *t*) denotes allele frequency as a function of location (*x*) and time (*t*), *s* denotes the selection intensity, and *σ*^2^ denotes the variance of the parent-offspring dispersal distribution (Eq 1):

$$\frac{\partial }{\partial t}p\left(x,t\right)=\frac{1}{2}\sigma^{2}\frac{\partial^{2}}{\partial x^{2}}p\left(x,t\right)+sp\left(x,t\right)\left(1-p\left(x,t\right)\right).$$ (1)

**Fig 2:**
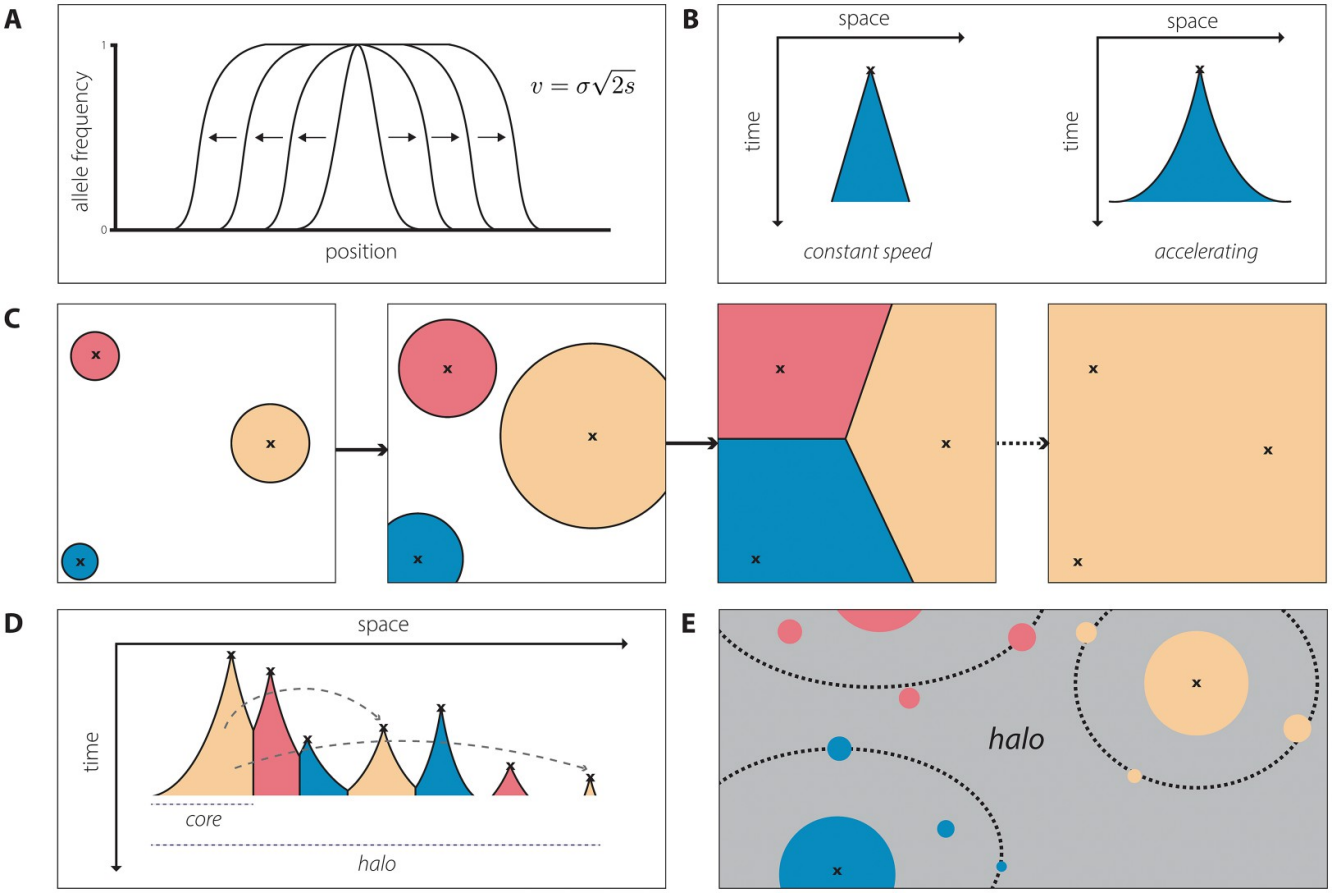
Summary of reviewed spatial models. (A) Schematic of Fisher’s wave of advance in one dimension [19]. (B) Space-time diagrams under regimes of constant vs. accelerating dispersal [22]. (C) Cartoon representation of parallel adaptation resulting in a tessellation-like pattern of genetic variation (as under the allelic exclusion assumption in [22]), followed by the later fixation of a single allele (as resulting, for instance, from a change in the adaptive landscape favoring a single allele present from standing variation). (D) Space-time diagram under the parallel exclusion assumption [22,23]. (E) Cartoon of the core-halo structure identified by [23] for spatial soft sweeps under long-distance dispersal.

A dynamic in this model is that a traveling wave (Fig 2A) can emerge where the adaptive allele expands through the surrounding population. The minimum velocity of the wave is determined by $v=\sigma \sqrt{2s}$ (Fig 2A). This finding underscores the intuition that an allele with a greater fitness advantage will spread more quickly through the population and that the general scale of dispersal (*σ*) in the population will also impact the speed of spread. The result also gives the more subtle insight that the dependence of velocity on *s* is less than linear: A variant that is twice as adaptive as a reference variant will spread at a rate only $\sqrt{2}$ times faster.

The wave of advance model—and other members of the general class of “reaction-diffusion” models that are related to it—have been a workhorse in mathematical biology, with applications that extend beyond the spread of adaptive alleles to ecological problems such as modeling the spread of invasive species [21,24] and the evolution of range limits (reviewed in [25]). For instance, reaction-diffusion equations can be extended to incorporate Allee effects, resulting in bistable equilibria affecting wave expansion (see [26,27]). In the realm of population genetics, the model continues to be studied theoretically—for example, a recent extension to address finite population size shows that the speed of spread for an adaptive allele is dependent on population density as well as dispersal and selection, with the wave of advance result corresponding to the maximal speed under infinite population size [28]. The wave of advance model has only occasionally been applied to empirical data (with relevant examples being [32–37]).

In standard reaction-diffusion models, a key limitation is that the representation of movement as a diffusion process implies that the distribution of dispersal distances (the dispersal kernel) must decrease with distance at least as quickly as an exponentially decaying function (i.e., the distribution has an “exponentially bounded tail” or more explicitly a “negative exponentially bounded tail”; Fig 1A). Mollison [38,39] showed formally that for dispersal kernels, which are not exponentially bounded, the upper velocity of the traveling wave is unbounded and will tend to infinity as time tends to infinity. This precludes the application of reaction-diffusion models to systems with substantial probabilities of long-distance dispersal events.

In the following sections, we describe extensions of spatial modeling to study cases with long-distance dispersal and multiple adaptive mutations (summarized in Table 1).

**Table 1:** Comparison of reviewed forward-in-time spatial population genetics models.

Model	Multiple Origins	Fat-Tailed Dispersal	Isotropic Dispersal	Stochastic Model	Heterogeneous Selection	Heterogeneous Dispersal
Fisher 1937 [19]	No	No	Yes	No	No	No
Kot et al. 1996 [8]	No	Yes	Yes	No	No	No
Ralph and Coop 2010 [22,29]	Yes	Yes	Yes	Yes	No	No
Hallatschek and Fisher 2014 [30]	No	Yes	Yes	Yes	No	No
Ralph and Coop 2015 [31]	Yes	No	Yes	Yes	Yes	No
Paulose et al. 2019 [23]	Yes	Yes	Yes	Yes	No	No

### The case with long-distance dispersal

In many biological systems, the assumption that the offspring dispersal process is diffusive is unlikely to hold due to the possibility of long-distance dispersal events. When the dispersal kernel is fat-tailed, with probability mass shifted away from intermediate values toward large values in the tail of the distribution, long-distance dispersal events become more common relative to short-distance dispersal events (Fig 1A). The idea that long-distance dispersal contributes to faster spread is intuitive and was considered formally over 120 years ago in the context of seed dispersal [40].

A framework for incorporating fat-tailed dispersal kernels was developed by Kot and colleagues [8] in a model of ecological invasions. In this model, the behavior of population density in the next generation is modeled by an integro-difference equation—a form of recurrence relation—involving a dispersal kernel, *f(r)* for a jump of length *r*, and the local population growth, *g(N)*, as a function of current population density *N*(*x*, *t*) (Eq 2).


$$N\left(x,t+1\right)=\int_{-\infty }^{\infty }\;f\left(x-x\text{'}\right)g\left(N\left(x\text{'},t\right)\right)dx\text{'}.$$ (2)

Two implicit assumptions in this model as well as Fisher-KPP models are that the dispersal kernel depends only on distance and that local growth is dependent only on the state of the current population. However, this model is more flexible with respect to the form of the dispersal kernel, making it more appropriate for modeling populations with long-distance dispersal. The results of Kot and colleagues [8] agree with the conclusions made by Mollison in that reaction-diffusion models may underestimate the speed of spread of an invasion (or, analogously, an adaptive allele) involving long-distance jumps and that the wave of advance can accelerate under fat-tailed dispersal kernels.

The underlying process of long-distance dispersal in a finite-size population is inherently stochastic, which may result in the inaccuracy of deterministic models when long-distance dispersal is frequent [30,41]. Ralph and Coop [22] show that under some conditions, properties of a stochastic traveling wave can be approximated well by considering a single “mean” wave that is representative of the wave’s path (their work in this area arises in the case of multiple adaptive lineages and is further described in the next section).

More generally, Hallatschek and Fisher [30] develop a fully stochastic characterization of long-distance dispersal in a model where the distribution of *successful* jumps as a function of distance, *f*_*s*_(*r*), has a power-law tail with the power-law exponent being determined by the number of dimensions, *d*, and a constant, *α*, such that lower values of *α* correspond to a more fat-tailed distribution. This approximation is derived from similar parameters as in previous models: *s* the strength of selection, $\hat{N}$ a population density constant, and *f(r)* the dispersal kernel (Eq 3):

$$f_{s}\left(x\right)=s\hat{N}f\left(r\right)\approx \frac{\epsilon }{x^{d+\alpha }},$$ (3)

Here, *ϵ* is approximately the proportion of successful jumps and *α* represents the combined effects of various factors contributing to the rate of long-distance dispersal.

Using an iterative scaling argument, the authors show that under a range of fat-tailed offspring distributions, the rate of spread of the adaptive allele can far exceed the classic wave of advance result. Further, varying the value of the power-law exponent in the dispersal kernel, (*d* + *α*), results in distinct regimes of asymptotic growth behavior. These regimes can be classified as linear growth (i.e., constant speed, as in the wave of advance) versus superlinear (i.e., accelerating) growth, the latter having jumps whose size is given approximately using a exponential, stretched exponential, or superlinear power-law distribution depending on (*d* + *α*). In the discussion, the authors indicate their model may be extended to consider infection dynamics within an epidemiological framework (specifically the SIR framework, defined later in this paper), noting how the passage of individuals between disease stages will alter the spreading velocity and lessen opportunities for long-range jumps.

### The case with multiple adaptive mutations

In a spatial context, the recurrent introduction of an adaptive allele in distinct populations is an important complicating factor. Multiple variants may arise and eventually compete within a range of interest, such as those resulting from multiple mutations or standing variation prior to a change in selective pressures [42–44].

The extension of the wave of advance model to the case of multiple adaptive alleles was considered by Ralph and Coop (Fig 2B and 2C). In their model, a key assumption is made in that the independently spreading types are neutral relative to each other (termed “allelic exclusion”). In addition, the model of Ralph and Coop [22] assumes that the first adaptive variant to reach a deme quickly rises to fixation (precluding local competition within a deme), that migration is weak such that this fixation event occurs prior to any further dispersal, and that there is no initial standing variation for the adaptive allele.

Using this model, the authors found that multiple adaptive variants arise and spread through the habitat until they encounter another adaptive allele (Fig 2B and 2C), eventually “tiling” the habitat. In the nomenclature of hard versus soft sweeps used in population genetics [42], this represents a *soft sweep* from the perspective of the total population as multiple variants spread as part of the response to selection; however, *a hard sweep* occurs at any single locale. If instead the selective advantage of one allele is higher than the others, then a single variant may potentially spread across the entire landscape (i.e., a hard sweep at the level of the total population; Fig 2C, right-most panel).

In the model of Ralph and Coop [22], if each wave is assumed to spread at a constant speed, as in the original wave of advance model, the expected distance traveled by a wave before encountering a different wave can be quantified by a compound parameter termed the “characteristic length” *χ*, which encapsulates the major properties the process in a way that should be useful across different geographies including, perhaps, more realistic ones than the homogeneous geography originally modeled. As the authors discuss, this concept is similar to the idea of an effective population size that can be obtained by several different combinations of model parameters. The exact form of this metric is given in (Eq 4), where *σ* and *s* represent the standard deviation of the dispersal distance distribution and the strength of selection, respectively, *λ* represents the rate of new mutations that eventually fix in the local population (per unit area per generation), and *ω*(*d*) is the area of a sphere of radius 1 in *d* dimensions:

$$\chi ={\left(\frac{\sigma \sqrt{2s}}{\lambda \omega \left(d\right)}\right)}^{1/\left(d+1\right)}.$$ (4)

Simulation results under a version of this model with long-distance dispersal show higher observed rates of parallel adaptation than predicted by the diffusive theory, further motivating the development of theory that simultaneously characterizes long-distance jumps and parallel adaptation. Notably, the interaction between expanding waves can only be analytically characterized in the case of constant speed, precluding comprehensive analysis of long-distance dispersal events or fat-tailed dispersal distance distributions, which violate the constant speed assumption. However, this framework is capable of describing dispersal under a more general scenario, in which constant speed is not assumed, using an alternate formulation of the characteristic length (we refer interested readers to [22] for additional details).

In later work by Ralph and Coop [29], the model is extended by allowing for standing variation in an adaptive allele prior to a change in selective pressures. The authors show that initial standing variation results in a greater probability of convergent evolution as well as a decrease in the time until adaptation of the global population, though adaptation is primarily local. Thus, temporal changes in selection (as could lead to selection on standing variation), in addition to dispersal, should be considered in the context of multiple circulating allelic types. Lastly, we note that recently developed methods for detecting parallel and convergent adaptation from genetic data (for instance, [45,46]) will be useful alongside theoretical modeling toward these aims.

### The case with multiple mutations and long-distance dispersal

In many applications, including SARS-CoV-2 evolution, both long-distance dispersal and parallel adaptation are relevant, necessitating a model incorporating both fat-tailed dispersal distributions and multiple mutations. The model in Paulose and colleagues [23] extends the iterative scaling argument for long-distance dispersal presented in Hallatschek and Fisher [30] to this case. Similar assumptions are made as in the model of Ralph and Coop [22], including an assumption that all mutations have the same selective advantage and that within-deme dynamics are irrelevant. Under this model, the spatial distribution of a single adaptive allele can be separated into two regions: a “core” region that spreads early on, before its spread is impeded by other clones, and a “halo” region of distant clusters seeded by long-distance jumps (Fig 2D and 2E).

In the case of soft sweeps with long-distance dispersal, the spatial dynamics are governed by a “mutation-expansion balance” because the spread of an advantageous allele can occur either via geographic expansion of an allele or through recurrent mutation. The framework presented by Paulose and colleagues [23] provides insight into both sides of this balance. For instance, in terms of dispersal, the authors find that in simulations with small values of the *α* parameter, i.e., more fat-tailed dispersal kernels (see Eq 2), early long-distance jumps are very important to the resulting spatial distribution of adaptive alleles. For higher values of *α*, the dynamics are more similar to the constant speed case described in Ralph and Coop [22]. Pertinent to considerations of mutational input, they additionally show that the number of independent origins of a mutation can be estimated using characteristic length scales introduced in Ralph and Coop [22] along with a derived characteristic time scale.

Notably, the core-halo structure has significant impact on the dynamics of soft sweeps and on their detection in genetic data. Under a fat-tailed dispersal kernel, local diversity is expected to be greater than under diffusive spread, but the growth rate of individual clones may be limited. Due to the assumption of allelic exclusion, core and satellite regions may never merge. As such, long-distance dispersal makes the detection of soft sweeps from global data more difficult, but also increases local diversity, making soft sweeps easier to detect in local samples. These results have clear applications for the interpretation of signals in genomic surveillance data.

Lastly, the closely related phenomenon of clonal interference [47]—which arises when the fate of a selected allele is affected by competition with other selected alleles—is highly relevant to the spread of adaptive alleles in asexually reproducing populations such as viruses. How clonal interference acts in spatial populations was studied by Martens and Hallatschek [48] in one and two dimensions. Clonal interference is more prevalent in spatially structured populations, though long-range migration can mitigate its effects. Given the inherent similarities between the processes of soft sweeps and clonal interference, greater integration between their distinct study may prove insightful toward advancing the understanding of the spread of adaptive alleles.

## Complications in adaptive evolution: Spatially heterogeneous selection, allele surfing, and adaptive landscapes

Many models cited above assume homogeneous selection across the entire geographic landscape; however, this is often untrue across the species range and so modeling heterogeneous selection is important. Differing selection along a cline has long been studied using reaction-diffusion models (see [49–52]) and in the context of integro-difference equations (for instance, [53]). Many relevant models of spatially varying selection also arise in the study of the evolution of quantitative traits [54–56], species range expansions and range limits (see, for instance, [57,58]), and discrete population or metapopulation models (see [59,60]). In the case of the spread of adaptive alleles, recent work has addressed the case where fitness is “patchy” across the geographic landscape, i.e., when certain alleles are adaptive in some local environments but deleterious in other regions, using stochastic approaches. For instance, an additional paper by Ralph and Coop [31] derives a critical distance between regions where the allele is favored, and this distance determines whether an allele is expected to evolve independently in each region or whether an influx of the adaptive allele from migration is expected. Notably, this model assumes that the dispersal displacement follows a Gaussian distribution and so may not be well suited for studying long-distance dispersal.

In studying alleles that appear to be adaptive, it is also important to consider processes that can result in neutral alleles appearing to be selected. In particular, allele surfing is a phenomenon in which alleles present at the edge of an expanding population drift to higher than expected frequencies [61,62]. These alleles may be neutral, adaptive, or even deleterious [63]. This phenomenon is a result of stochastic effects at the wave front, akin to serial founder effects, such that mutations close to the edge in effect produce more offspring than those occurring internally. Notably, after the expansion has occurred, the center of the spatial distribution of a “surfed” allele will often be distant from its point of origin, complicating the interpretation of frequency data [61]. Allele surfing results in distinct regions where an allele is carried at high frequency, which radiate from the allele’s mutational origin along the direction of an expansion, referred to as sectors [64]. This lowers local genetic diversity, resulting in a pattern that may be misinterpreted as evidence of a selective sweep. Long-distance dispersal events are capable of breaking down these regional patterns and maintaining local diversity, though the behavior of these systems is dependent on the extent of long-distance dispersal, i.e., the tails of the dispersal kernel [65]. The offspring distribution of alleles at the wave front is also overdispersed due to the occurrence of these chance events, violating the assumptions of standard population genetics models (see [66,67] for recent work in this area). Allele surfing dynamics are potentially relevant to quickly expanding viral populations, such as SARS-CoV-2 VOCs [68,69], and introduce further complexities to the interpretation of mutation frequency data.

Lastly, the models discussed thus far largely disregard the process of mutational introduction—a topic that can be discussed in terms of a nongeographic “landscape” of mutational or sequence space. Characterizing adaptive landscapes allows one to address questions regarding the number of possible adaptive variants accessible via mutation, including the number and characteristics of paths leading to them, and the probability of each occurring. Describing a full adaptive landscape is extremely difficult due to the large number of mutational combinations and orderings of paths, though important insights can be made by focusing on subsets of relevant mutations (as in [70]) or through approaches such as deep mutational scanning (see [71] for such analyses in SARS-CoV-2).

## Limitations of existing models in the context of SARS-CoV-2 evolution

### Dispersal approximations in the context of SARS-CoV-2 evolution

In the epidemiological literature, models of pathogen spread often use empirically driven paramaterizations of dispersal that are temporally and spatially varying based on air traffic patterns [72,73], commuter data [74–76], mobile phone data [77,78], for example. However, the theoretical population genetic models reviewed here assume simple models of dispersal. The classic wave of advance model—with its assumption of short dispersal distances—is clearly not appropriate for modeling SARS-CoV-2 due to the frequency of long-distance plane travel before and during the epidemic, with similar limitations arising for other respiratory pathogens (including SARS [79] and influenza [80]) in which air travel has a significant role in transmission. Population genetic models that do explicitly account for long-distance dispersal (including [23,30]) assume that the probability of a jump can be described a function of its distance alone and do not address how connectivity patterns are not simply a function of distance (for instance, Fig 1C) and can vary over time. In addition, none of the continuous models address the irregular boundaries imposed by finite habitats (“edge effects”), which can act to constrain the spread of an adaptive allele [81]. Including such edge effects may be necessary to understand the speed of spread in irregular habitats. Future genetic models explicitly accounting for complex dispersal patterns, informed by empirical mobility data, may be essential to accurately model SARS-CoV-2 and other modern-day infectious diseases, for the above stated reasons as well as the discrete nature of data collection for viral sequencing. We note that the relevance of long-distance dispersal is inherently connected to its mode of transmission, with nonrespiratory viruses such as Ebola [82] or pandemics occurring before modern air travel [83] plausibly depending more so on physical distance.

Emerging research on COVID-19 dynamics emphasizes the importance of complex models of dispersal. For instance, mobility networks reconstructed from mobile phone data suggest that even during the lockdown phases of the pandemic, the majority of infections derive from a minority of spatial locations [78]. Using retrospective phylogeographic approaches, it has also been shown that asymmetric migration out of a large urban area (London) has contributed to accelerated dispersal rates of VOCs in tandem with adaptive traits such as increased transmissibility [68]. Together, these studies emphasize the role of temporal changes in dispersal behavior as well as spatially heterogeneous dispersal patterns affecting human mobility in the adaptive evolution of SARS-CoV-2.

One promising approach to this problem is presented by Brockmann and Helbing [84], in which physical distance is replaced by an “effective distance” derived from a mobility network and then used in a reaction-diffusion model coupled with a stochastic SIR spreading process. Formally, the effective distance between directly connected nodes *m* and *n* in a network is defined as *d*_*mn*_ = (1 − log*P*_*mn*_), where *P*_*mn*_ is the probability of travelers leaving from node *n* moving to node *m* in a time step (the distance between nonconnected nodes is given by computing the effective distances of the shortest path between them). This is distinct from the context of resistance distances used in ecological and genetic models, which are based on the length of a random walk between two nodes (see [85] for a review). In particular, while resistance distances (and most distance metrics, generally) are symmetric, and this can be problematic (for example, [86]), effective distances can be asymmetric. Recently, effective distances have been used in tandem with empirical mobility networks (based on mobile phone data) to measure the impact of specific travel behaviors and policies on COVID-19 spread [87]. By decomposing mobility into subcomponents representing distinct modes of travel, the authors identify an increase in variance in the distribution of effective distances during lockdown periods, thus emphasizing the role of temporally heterogeneous dispersal in COVID-19 spread. Thus, effective distance measures have potential for use in genetic models as a means to incorporate complex network structures in human mobility.

### Selection approximations in the context of SARS-CoV-2 evolution

The bulk of models of the spread of adaptive alleles assume selection is homogeneous across the landscape (Table 1). Yet, heterogeneous selection is likely to be an extremely important aspect of SARS-CoV-2 evolution due to geographic variability in immunity (for example, due to previous infection, vaccination, and/or uptake of other public health and medical interventions) as well as variation in frequencies of other VOC lineages. These dynamics induce a form of negative frequency-dependent selection. Accordingly, substantial variation in the inferred selective advantage of several VOCs across countries has recently been identified from sequencing data [88].

There is also mounting evidence that variation in the adaptive landscape is important to SARS-CoV-2 VOC evolution. For instance, signatures of selection in SARS-CoV-2 sequencing data are consistent with a major shift in the SARS-CoV-2 adaptive landscape in the fall of 2020, such that mutations in the receptor binding domain of the N501Y lineages gained a detectable fitness advantage only after this shift [89]. Additionally, mutations have been observed in SARS-CoV-2 sequence data that may be responsible for increased mutagenesis coinciding with the appearance of VOCs [90]. Even if the adaptive landscape is too complex to quantify, the metaphorical comparison remains useful as a theoretical tool and for framing observations of recurrent adaptive mutation.

Furthermore, existing models that allow for the simultaneous spread of multiple adaptive lineages assume that the adaptive alleles are relatively neutral with respect to each other [22,23]. This precludes the case of competing variants with differing fitness (for example, in SARS-CoV-2, the Alpha variant versus the Wuhan strain in early 2020 and the Delta and Omicron variants of late 2021 and early 2022). As such, further work in this area is critical to the study of origins and dispersal of adaptive VOCs in this context, as well as for the study of other geographically widespread species and pathogens.

In closing this section, we emphasize that future work addressing the limitations of existing models—especially those pertaining to heterogeneous dispersal, spatially and temporally varying selection, and multiple adaptive lineages, as discussed in this section—remains paramount, both for epidemiologically relevant questions and broader applications in biology that depend on understanding the rate at which novel adaptive variants spread.

### Incorporating epidemiological dynamics: SIR-type models

Besides the spatial and evolutionary issues raised in the above sections, models originating from the population genetics literature do not account for phenomena specific to infectious disease. There are two particularly relevant aspects of these dynamics: the varying proportion of individuals in the population in particular disease states such as susceptible, exposed, infectious, or recovered (as addressed by compartmental models such as SIR models) and the impact of “superspreading” events on variant evolution.

**Fig 3:**
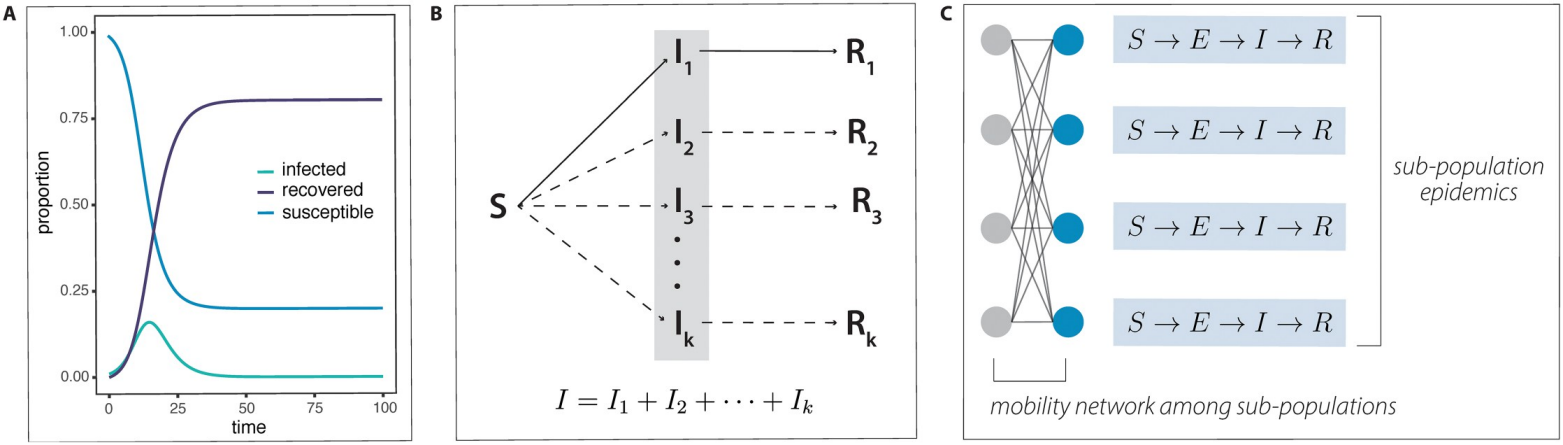
SIR models. (A) Proportions of susceptible, infected, and recovered individuals over time for an infection rate of 60% and a recovery rate of 30% per generation in a standard SIR model, as in Kermack and colleagues [91]. (B) Multistrain SIR model, as in Gordo and colleagues [93]. *I*_1_, …, *I*_*k*_ represent infected infections with strains 1, …, *k*. (C) SEIR (Susceptible Exposed Infectious Recovered) model with population substructure, as in Chang and colleagues [78].

The simplest model for the dynamics of epidemic spread is the SIR model, in which, at any given time, each individual in the population is either susceptible, infected, or recovered/removed (Fig 3A; [91]). Basic SIR models assume that the pathogen does not evolve over time and that the population is well mixed. However, extensions to the SIR model exist, which can incorporate adaptive variants as well as population structure (see closely related “meta-population” models as discussed in [92]), both of which are essential to modeling SARS-CoV-2 evolution.

Multistrain versions of SIR models (such as [93–97]) can incorporate mutations, and as a result link epidemic dynamics to genetic diversity (Fig 3B). SIR models and extensions have also been incorporated as components into larger models of host-pathogen evolution (for instance, [98]), as well as spatial models (see [99–101]). As mentioned above, SIR models can be stratified by population and overlaid onto mobility networks in order to incorporate population structure (Fig 3C; [77,78]). The framework of Hallatschek and Fisher [30] (discussed above) can also be adapted to SIR models—as they discuss in their paper, the length of the infectious period can have important impacts on the spatial spreading of an epidemic (and vice versa).

### Incorporating epidemiological dynamics: Superspreading

**Fig 4:**
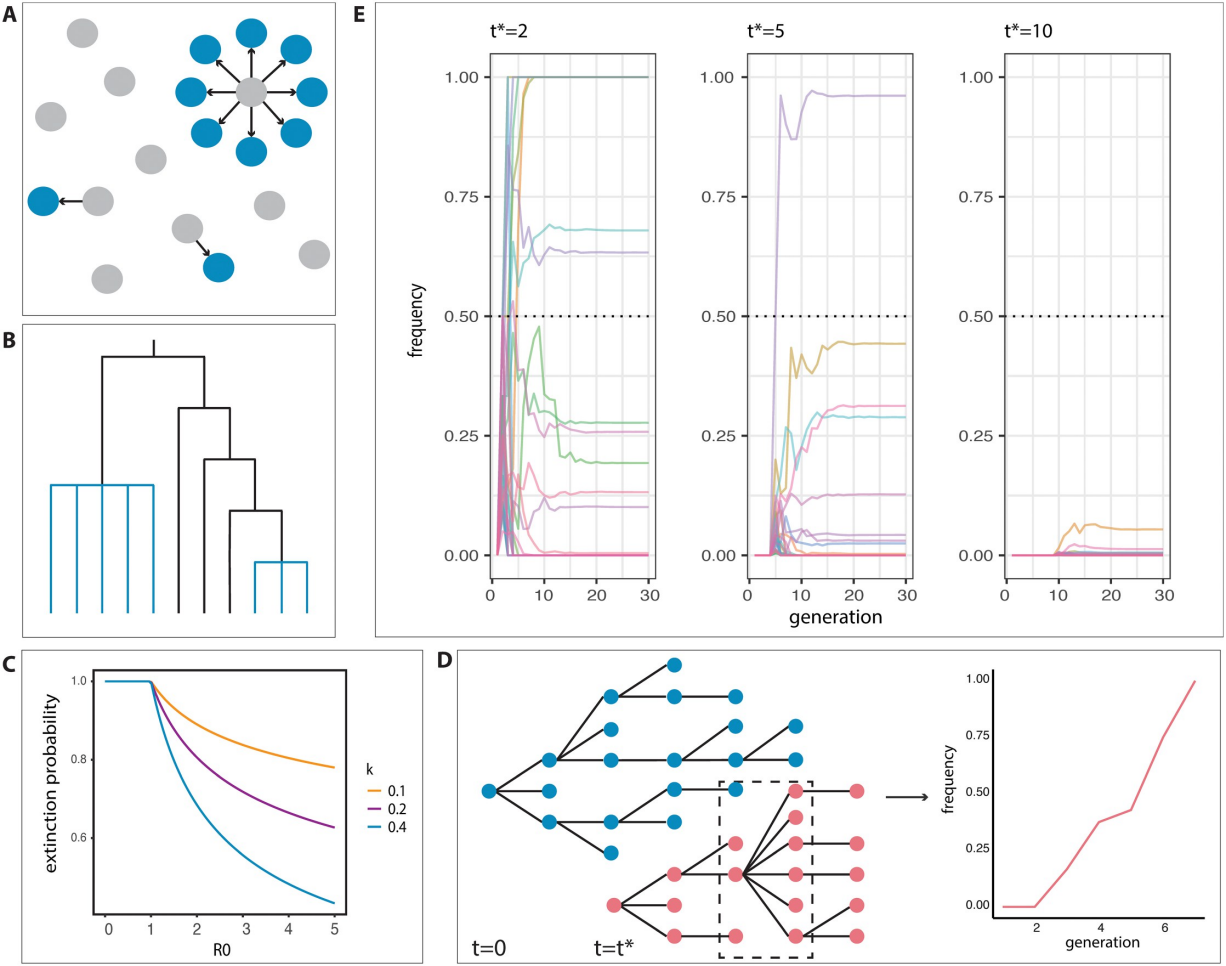
Effects of superspreading events on allele frequency trajectories. (A) Example of overdispersion in secondary case numbers. In this scenario, 10% of infected individuals in a previous generation (grey) give rise to 80% of new cases (blue), as has been reported for SARS-CoV-2 transmission. (B) Example of a simple multiple-merger coalescent, with multiple-merger events highlighted in blue. (C) Extinction probabilities for a negative binomial branching process with parameters *R*_0_ ∈ [0,5], *k* ∈ {0.1,0.2,0.4}. Probabilities were evaluated numerically as the solution to *g*(*s*) = *s* using the expression for the probability generating function *g(s)* as given in [12]. (D) Schematic of a multitype branching process model, with an initial type (blue) as well as an introduced type beginning at time t* (red). An example of a superspreading event is shown in the black dashed box. The plot on the right shows the resulting frequency trajectory of the introduced type. (E) When both types have a negative binomial offspring number distribution with identical parameters (i.e., the neutral case), the frequency of replacement by the introduced type is dependent on the introduction time t*. Simulations are of a two-type negative binomial branching process with introduction of the second type at t*. Both types have parameters *R*_0_ = 2 and *k* = 0.4. Simulations were run 100 times for 30 generations each, with only runs in which the initial type is nonextinct at t* visualized.

In the case of highly contagious viruses such as SARS-CoV-2, the distribution of the number of resulting secondary cases per infected individual can vary on an individual basis due to superspreader events (SSEs), in which one individual infects significantly more individuals than is expected (i.e., the number of secondary infections is much greater than the basic reproductive number, *R*_0_). Both contact tracing [102] and genetic [5,103] lines of evidence have established the role of SSEs in SARS-CoV-2 transmission routes, with analyses of case counts from February to May 2020 in the United States [104] and globally [105,106], suggesting that up to 80% of SARS-CoV-2 transmission events could be attributed to as few as 2% to 10% of infected individuals (Fig 4A). The resulting overdispersion in the number of secondary case numbers—the “offspring distribution”—can have significant downstream effects on the behavior of the epidemic, especially during periods of low case number [107]. In particular, overdispersion affects the probability that the epidemic eventually dies out on its own and has been implicated in patterns of VOC emergence and evolutionary success [5,108]. Notably, superspreading has also been shown to result in a fat-tailed distribution of secondary cases [109]. Superspreading behavior has been previously observed in several other epidemics, including outbreaks of SARS [12], MERS [110], and tuberculosis [111].

In the context of population genetics, we note that overdispersion in individual offspring number violates the assumptions of many population genetics frameworks, potentially biasing statistical inference under the assumptions of the Wright–Fisher model and the Kingman coalescent [112–116]. While it is possible that standard Wright–Fisher dynamics may still be appropriate in a large population size limit, it is unclear to what extent the Wright–Fisher diffusion and Kingman coalescent will apply for finite populations over short timescales (as is most relevant for understanding the short-term spread of advantageous variants). In simulations, it has been shown that uncorrected skew in offspring number—such that the offspring of single individual accounts for 5% to 10% of the next generation—results in incorrect inference of selective sweeps [116]. For coalescent modeling, rather than coalescent events only involving one pair of ancestral lineages at a time, “multiple-merger events” (Fig 4B) can occur. Techniques to account for multiple-merger events in the coalescent (Fig 4B; [114,115,117–120]), as well as more recent forward-time approximations, which incorporate selection [67,121], provide new avenues to move forward in this area. One relevant approach is the spatial Λ-Fleming-Viot model introduced in [122], which explicitly incorporates large fluctuations in reproductive success within a model of spatial structure.

In the epidemiological context, a branching process framework for studying SSEs was developed by Lloyd-Smith and colleagues [12] in response to the SARS epidemic of the early 2000s. Briefly, the number of secondary infections per case is drawn from a negative binomial distribution with mean *R*_0_ and dispersion parameter *k*, such that smaller values of *k* correspond to higher overdispersion and a higher probability of epidemic extinction (Fig 4C). This model can be extended to multiple variants by using multitype branching processes, as shown in Fig 4D.

One interesting possibility is that SSEs may lead to rapid fluctuations in a variant’s abundance, even in the absence of a selective advantage. As a simple exploration of this concept, we carried out simulations from a multitype branching process, in which a novel variant type has the same fitness as the ancestral type (a “neutral variant” Fig 4E). When overall case numbers are low (for instance, early in an epidemic or late but in a location where the numbers of infecteds have receded), a new neutral mutation can increase in numbers faster than the ancestral type population if it happens to be spread by several SSEs. This could potentially lead to the mistaken conclusion of selection favoring the novel variant in the case that the model is not well calibrated to the case of SSEs. The confusion would be akin to positive selection being mistakenly inferred during range expansions due to allele surfing (see “Complications in adaptive evolution” section). However, we note that this question is largely unresolved, and so further theoretical work is necessary to evaluate the limitations of population genetic models under superspreading regimes in practice.

These simulations point to several key possibilities regarding SSEs and variant evolution, namely (i) how early SSEs may result in large increases in neutral variant frequency and (ii) the role of variant introduction time in the effect of SSEs on frequency trajectories. Moving forward, more complex models that include spatial dynamics would be necessary to make conclusive statements regarding overdispersion’s global-scale effects on variant evolution for SARS-CoV-2 and other infectious agents.

Lastly, we note that neither population genetic models nor the branching process framework described above account for temporal autocorrelation in offspring number that is distinct from fitness effects, i.e., correlation between offspring number of infected individuals near one another in a transmission chain. For instance, if individuals tend to associate with others who have similar levels of precaution or risk perception, the number of secondary infections resulting from successive transmission events could show a positive correlation that is not related to any fitness characteristic of the virus. Additionally, if risk-taking or cautionary behavior is tied to geographic location (i.e., due to local government policies or lockdowns), there is further potential for spatial autocorrelation effects that are independent of viral fitness. Intriguingly, a recent analysis of SARS-CoV-2 transmission networks observed that individuals infected via superspreading tend to be a “superspreader” themselves more often than would be expected by chance, a phenomenon that would be consistent with the above described autocorrelation effects [123]. These possibilities further complicate theoretical modeling of superspreading behavior.

## Discussion

In considering population genetic models for the spatial spread of adaptive alleles and their potential applications to SARS-CoV-2 variant evolution, we have identified several shortcomings of the models with respect to both evolutionary and epidemiological complexities. These include aspects of both geographic dispersal (i.e., simultaneous short- and long-distance dispersal, dependence of spread on heterogeneous travel networks) and transmission or reproduction of the virus itself (i.e., superspreading). Beyond what we review above, one must also consider details of viral life history, such as how viral fitness is mediated through components of immune evasion and transmissibility [124], as well as the properties of the human adaptive immune system as an evolutionary system in and of itself [125]. For instance, the phenomenon of accelerated SARS-CoV-2 within-host evolution in immunocompromised individuals [126,127] has been recently discussed in the context of the Omicron variant, which carries an exceptionally high number of derived mutations [69].

Our review thus highlights several goals for future work (Table 2). An important strategic challenge is how to address them. The computational epidemiology literature includes many large-scale, parameter-rich models (for instance, the CityCOVID model from Argonne National Laboratory; [128]). Phylodynamic and phylogeographic methods take a retrospective approach (see, for instance, [68,129,130]). The theoretical population genetic literature (reviewed here) tends to be more abstract and prospective. Certainly, an integrative model involving spatial, genetic, and epidemiological aspects of SARS-CoV-2 evolution would be ideal, in principle, for developing better prediction and insight regarding the evolution of viral pathogens such as SARS-CoV-2. That said, more elaborate models pose an incredible technical challenge to develop. Even if achievable on a technical level, there is an inherent risk of failure given the vagaries of human behavior, including the unpredictable ways humans have responded to policy changes.

**Table 2:** Ongoing challenges for future work in theoretical models of the spread of adaptive viral variants.

Spatial models for the spread of adaptive alleles	Dispersal on heterogeneous networks Temporal variation in dispersal dynamics Consequences of a high variance offspring distribution
General processes of adaptive evolution	Structure of adaptive landscapes Models of multiple adaptive variants with varying fitness
Pathogen evolution	Integration of genetic and epidemiological modeling frameworks for infectious agents The impact of within-host evolution on transmissibility between hosts

Yet, work toward addressing the complexities listed in Table 2—either independently or in tandem—remains a worthwhile goal. Through rigorous spatial modeling, qualitative aspects of the possible evolutionary dynamics of viruses like SARS-CoV-2 will likely become apparent that can help guide public health responses. For instance, already, the core-halo structure identified in Paulose and colleagues [23] is insightful when interpreting observations of new clusters of variant transmission (see above). As a second example, the initial success of effective distance as a metric for simplifying models for the spread of SARS-CoV-2 [84,87] suggests the metric may be useful for genetic models. As a graph-based metric, its efficacy is also an indicator of opportunities to utilize results from the more general literature on spreading processes on networks [131–133]. In general, by studying these models and the patterns they predict—in particular those which are unexpected or perhaps counterintuitive outside of a spatial context—we may learn principles that will aid in the management of adaptive variants in future epidemics and pandemics.

Overall, improved modeling of these processes has the potential to answer many compelling questions regarding SARS-CoV-2 and future pandemics, for example, how often will novel adaptive variants spread only locally versus globally? What kind of lag time should we expect between origin in one location and arrival in another? How much interference should we expect between adaptive variants? And how is this impacted by the geographic location of origin of new variants and/or patterns of long range dispersal? Moreover, what is the relative importance of public health measures that control local transmission (for example, mask policies) versus host movement (for example, travel bans)?

In closing, our review of population genetic models for the spatial spread of adaptive variation identifies major gaps, in particular with respect to spatially and temporally varying dispersal, high variance in offspring number, and simultaneously spreading adaptive lineages. While we have largely focused our discussion on practical applications to modeling SARS-CoV-2 VOCs, the requisite development of theory will advance spatial genetic modeling generally. Beyond preparing for modeling and reacting to future epidemics, continued work in this area will give insights to problems in ecology and evolutionary biology such as the spread of invasive species and the consequences of population structure for adaptive evolution.
